# The Role of Specialty Palliative Care in Elective Surgical Oncology: A Systematic Review

**DOI:** 10.1245/s10434-025-17076-4

**Published:** 2025-02-25

**Authors:** Amanda K. Walsh, Marissa Z. Guo, Thomas Leuschner, Aslam Ejaz, Timothy M. Pawlik, Sharla Wells-Di Gregorio, Brittany Waterman, Jordan M. Cloyd

**Affiliations:** 1 Department of Surgery, The Ohio State University Wexner Medical Center, Columbus, OH USA; 2 Department of Surgery, Division of Surgical Oncology, University of Illinois Health, Chicago, IL USA; 3 Division of Palliative Medicine, Department of Internal Medicine, The Ohio State University Wexner Medical Center, Columbus, OH USA; 4 Division of Surgical Oncology, The Ohio State University Wexner Medical Center, Columbus, OH USA

**Keywords:** Supportive care, Surgical resection, Cancer surgery, Palliative medicine, Quality of life

## Abstract

**Background:**

Unlike advanced cancer populations, for whom early and routine specialty palliative care (PC) referral has demonstrated clear benefits for quality of life and symptom control, evidence supporting PC for patients undergoing curative-intent cancer surgery has been inconclusive.

**Method:**

A systematic review of the PubMed, Embase, Cochrane Library, and MEDLINE databases was performed to identify all studies evaluating the role of PC for patients undergoing curative-intent surgery for cancer.

**Results:**

Among the 12,886 publications initially retrieved, 14 met all inclusion criteria: two cross-sectional studies comprised of physician surveys, four cohort studies, five qualitative studies, one prospective trial, and two randomized controlled trials (RCTs). In non-randomized studies, PC was associated with increased advanced care planning and symptom control; however, both the PERIOP-PC and SCOPE RCTs found no significant improvement in quality of life with routine perioperative PC among patients undergoing major abdominal cancer surgery. Qualitative studies characterized patient experiences and physician perspectives of PC.

**Conclusion:**

This systematic review summarizes the existing literature on the role of PC in surgical oncology, highlighting the unique considerations and unmet needs of surgical patients that differ from those with advanced cancer. While the routine use of perioperative PC for patients undergoing cancer surgery is not supported, future research should focus on identifying high-risk patients who would most benefit from specialty PC and innovative methods of delivering supportive care in surgical oncology.

Palliative care (PC) is an interdisciplinary approach aimed at improving quality of life (QOL) for patients facing serious illness by focusing on symptom management, psychosocial support, and the alignment of treatment goals with patient preferences. While traditionally associated with end-of-life care, specialty PC is now increasingly recognized as an important component of multidisciplinary care throughout the disease trajectory, especially for patients with intricate medical needs and high symptom burden, such as those diagnosed with cancer.^1–4^ Multiple studies have documented the benefits of PC involvement in cases of advanced cancer, including reduced symptom distress, enhanced psychological well-being, higher quality communication, increased patient and family satisfaction with care, and reduced health care costs.^1,5–9^ As a result, early integration of PC into standard oncologic care is now recommended by numerous guidelines.^10–13^

The role of PC for patients with early-stage cancer undergoing curative-intent surgery is less clear. In theory, early PC consultation can provide valuable support by addressing the needs specific to this population, such as managing postoperative pain and facilitating decision making around treatment options. Palliative services may also be important for long-term survivorship concerns of patients who have completed curative-intent surgeries and experience chronic symptoms and diminished QOL as a result.^14^ Nevertheless, the integration of PC into surgical oncology has been variable and underutilized, and many patients do not receive timely or adequate palliative support.^15,16^ Moreover, the literature on the empiric benefits incorporating PC in surgical oncology, particularly for patients receiving curative-intent therapies, is sparse.^17^ In such cases, the efficacy and optimal timing of PC consultation remains unclear. This gap highlights the need for a comprehensive appraisal of existing studies to identify best practices, potential barriers, and areas requiring further research surrounding the provision of PC to surgical oncology patients.

This systematic review sought to evaluate the current evidence on specialty PC consultation for patients undergoing oncologic surgery. Specifically, we aimed to explore factors that influence the uptake and timing of PC referral in this population, as well as its impact on patients’ QOL and healthcare utilization. By synthesizing the published research, this review seeks to inform future clinical practice, ultimately supporting a more integrated and patient-centered approach to surgical oncology.

## Methods

### Search Strategy and Study Selection

A literature search of the PubMed, Embase, Cochrane Library, and MEDLINE databases was performed using the following Medical Subject Heading (MeSH) search terms: *palliative medicine*, *palliative care*, *palliative care nursing*, or *hospice*; *neoplasm*, *carcinoma*, *adenocarcinoma*, *cancer*, *malignancy*, or *sarcoma*; *surgery*, *surgical procedures*, *operative*, *resection*; and *curative intent*, *cure*, *early stage*, *complete resection*, *elective*, or *non-metastatic*. Multiple combinations of search terms were used, and both free text and controlled vocabulary (MeSH and Emtree) were included. The initial search was completed on 24 June 2024.

All studies that examined patients undergoing curative-intent surgery for solid organ cancers were included. Cancer operations for palliative purposes were excluded. PC was restricted to specialist palliative medicine delivered by a secondary care provider and did not include acute and chronic pain services, psychosocial oncology, chaplaincy, psychological services, hospice, and end-of-life PC. Case reports and case series (*n* < 5), study protocols, review articles, commentaries, letters, and narratives were excluded. Abstracts were allowed if there were no full-text duplicates. No restrictions were placed on date of publication. Additional relevant studies were identified through manual searches of the literature.

Initial screening of eligible studies was performed through a review of titles and abstracts and followed by a secondary screening of the full text. Each phase of screening was conducted by two independent reviewers, and disagreements were settled by a third reviewer. The literature search and screening were completed using Covidence (Melbourne, VIC, Australia). The systematic review was performed according to the Preferred Reporting Items for Systematic Reviews and Meta-Analyses (PRISMA) guidelines. This was not a registered study.

### Data Extraction

Data extraction from the included studies was undertaken by one author (AKW). Variables that were extracted included publication year, study period, country of origin, study design, cancer types, primary and secondary outcomes, and timing of PC involvement. A meta-analysis was not completed due to anticipated small sample sizes and between-study heterogeneity.

### Quality Appraisal

Each study was assessed for quality and presence of bias using the Joanna Briggs Institute (JBI) Critical Appraisal checklists. The JBI checklists for cross-sectional,^18^ cohort,^18^ qualitative,^19^ quasi-experimental (non-randomized),^20^ and randomized controlled trials (RCTs)^20^ contain a series of ‘yes’ or ‘no’ questions to evaluate study design. Cut-offs at 70% and 50% of questions that were answered ‘yes’ were used to determine high- and medium-quality studies, respectively. No studies were omitted based on quality assessment to ensure the entire body of literature was represented in this review.

## Results

### Study Characteristics

**Fig. 1:**
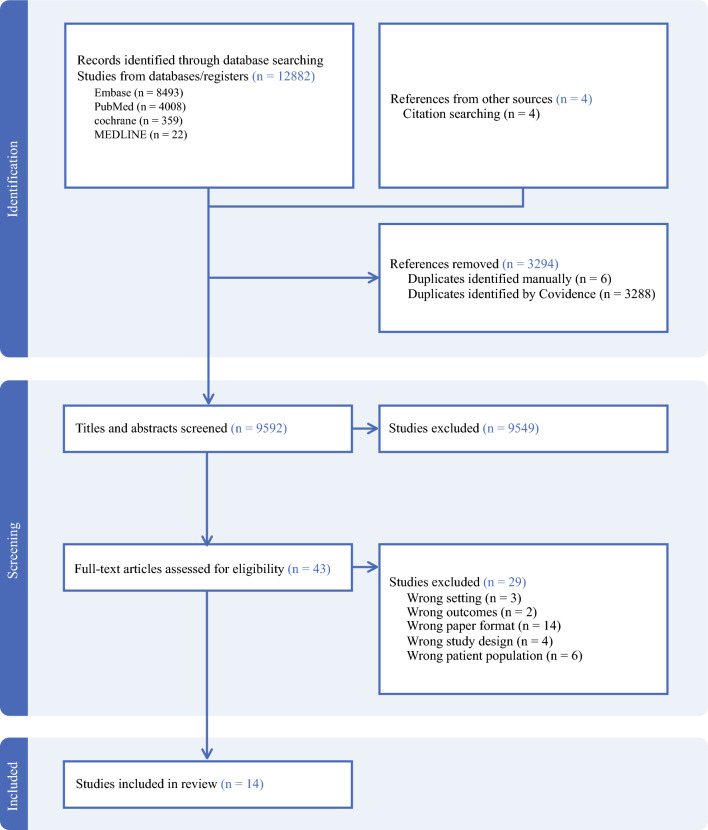
Study selection and PRISMA flow diagram. PRISMA preferred reporting items for systematic reviews and meta-analyses

Among the 12,886 publications retrieved in the initial query, including 4 from searching the reference list of relevant studies, 3294 were identified as duplicates. Title and abstracts were screened for 9592 studies. After a full-text review of 43 articles, 29 were deemed ineligible for inclusion. Ultimately results were extracted from 14 studies that met the inclusion criteria (Fig. 1). The literature search identified two cross-sectional studies comprised of physician surveys,^21,22^ four cohort studies,^23–26^ five qualitative studies,^27–31^ one non-randomized trial,^32^ and two RCTs^33,34^ (Table 1). Of the 14 studies included, two were abstracts.^22,27^ Three qualitative studies were nested in the Surgery for Cancer with Option of Palliative Care Expert (SCOPE) trial,^29–31^ and one was nested in the Perioperative Palliative Care (PERIOP-PC) trial.^28^ Most studies were performed in the United States (*n *= 12), with one that included international participants in a physician survey.^22^ The other countries represented were Sweden (*n* = 1) and Taiwan (*n *= 1). Gastrointestinal malignancies were the most commonly reported cancer type. Overall, 6876 patients and 287 physicians were the subjects of the included studies.

**Table 1:** Study characteristics for included trials

First author	Publication year (data collection)	Country	Study type	Cancer type	Sample size (study population)	Study methods	Timing of PC involvement	Primary outcome
Adolfsson^21^	2022 (2016–2017)	Sweden	Physician survey	Mixed	130 (C: 70; I: 60)	Comparison of surgical specialties with medical specialties	Varies by specialty	Attitudes, practices, and experiences of physicians regarding the referral process, integration, and transition between oncologic care and PC
Aslakson^33^	2023 (2018–2020)	USA	RCT	Upper gastrointestinal cancers	359 (C: 177; I: 182)	Patients co-managed with PC and surgeon team compared with usual care plus NCCN PC recommendations. Opportunity for one caregiver to participate	Before surgery and 1 week, 1 month, 2 months, and 3 months after surgery	QOL (FACIT-PaL)
Bansal^23^	2023 (2016–2022)	USA	Retrospective cohort	Advanced abdominal and soft tissue malignant tumors	326 (C: 182; I: 144)	Rates of ACP before and after EHR designation work flow implementation	New patient visit at surgical oncology clinic	AD designation and documentation
Chan^24^	2023 (2010–2017)	USA	Retrospective cohort	Breast cancer	5496	Referrals to and utilization of palliative medicine and other ancillary services after breast cancer surgery	After surgery	Referral to and use of ancillary service
Chen^32^	2024 (2020–2022)	Taiwan	Prospective cohort study	Head and neck cancer	82 (C: 13; I: 69)	Patients with recently diagnosed HNC with plans to treat that did not agree to early PC compared with those who agreed	At trial enrollment (within 8 weeks of HNC diagnosis), twice at 1- and 3-month timepoints. Monitored by the EPC team for 12 months after enrollment	DT, MDASI-T, EORTC QLQ-C30
Demyan^22^	2022 (2021)	USA	Physician survey^a^	GI cancers	138	Surgeons who perform curative-intent pancreas cancer surgery surveyed on the role of preoperative PC consultation	Before surgery	Characteristics associated with identifying potential benefit of preoperative PC consultation and co-management
Guo^25^	2023 (2011–2021)	USA	Retrospective cohort	Mixed	344 (C: 288; I: 116)	Rates of PC referral for patients undergoing curative-intent surgery unexpectedly aborted	After aborted cancer surgery	Surgical indications, clinicopathologic characteristics, patient outcomes, and PC involvement
Hartman^27^	2023	USA	Qualitative^a^	Pancreas	19 (C: 9; I: 10)	Opinions of high-volume pancreas surgeons compared with PC physicians	Not reported	Role of specialty PC in patients with potentially curable pancreatic cancer
Holdsworth^28^	2024 (2019–2021)	USA	Qualitative	Patients enrolled in PERIOP-PC	23	Qualitative study of patient experience for the intervention arm of PERIOP-PC	Before surgery and 1 week, 1 month, 2 months, and 3 months after surgery	Experiences of patients assigned to perioperative PC
Rodriguez^26^	2021 (2016–2019)	USA	Retrospective cohort	Not reported	32 (C: 26; I: 8)	Comparison of patients treated or considered for treatment with CRS-HIPEC who received PC consultation versus those who did not	Not reported	Identify potential variables affecting a clinician’s decision to consult PC
Shinall^30^	2021 (2018–2021)	USA	Qualitative	Patients enrolled in SCOPE	50	Review of PC documentation for first 50 patients enrolled in the intervention arm	Before surgery	Types of psychosocial stressors facing patients preoperatively
Shinall; Ely^29^	2023 (2019–2021)	USA	Qualitative	Patients enrolled in SCOPE	47 (C: 21; I: 26)	Qualitative interview of first 50 patients enrolled in the intervention arm	Before surgery, twice weekly inpatient after surgery, three visits before postoperative day 90, and during any inpatient readmission	Experiences of patients assigned to perioperative PC
Shinall; Martin^34^	2023 (2018–2021)	USA	RCT	Abdominal cancers	235 (C: 117; I: 118)	Patients scheduled for curative intent cancer surgery seen by PC in the perioperative period versus usual care	Before surgery, twice weekly inpatient after surgery, three visits before postoperative day 90, and during any inpatient readmission	Physical and functional QOL (FACT-G)
Williams^31^	2024 (2019–2021)	USA	Qualitative	Patients enrolled in SCOPE	47 (C: 21; I: 26)	Qualitative interview of first 50 patients enrolled in the intervention arm	Before surgery, twice weekly inpatient after surgery, three visits before postoperative day 90, and during any inpatient readmission	Experiences of patients assigned to perioperative PC

^a^Abstract only

*C* control, *I* intervention, *QOL* quality of life, *PC* palliative care, *FACIT-Pal* Functional Assessment of Chronic Illness Therapy-Palliative Care, *ACP* advance care planning, *AD* advance directive, *EHR* electronic health record, *HNC* head and neck cancer, *DT* distress thermometer, *MDASI-T* MD Anderson Symptom Inventory, *EORTC-QLQ-C30* European Organization for Research and Treatment of Core Quality of Life, *CRS- HIPEC* cytoreductive surgery and hyperthermic intraperitoneal chemotherapy, *PERIOP-PC* Perioperative Palliative Care trial, *SCOPE* Surgery for Cancer with Option of Palliative Care Expert, *FACT-G* Functional Assessment of Cancer Therapy-Gen, *RCT* randomized controlled trial, *NCCN* National Comprehensive Cancer Network, *EPC* early palliative care, *GI* gastrointestinal

### Randomized Controlled Trials

Two RCTs were recently published examining the impact of specialist PC referral on outcomes of cancer patients undergoing curative-intent surgery. Although neither study was blinded to the patients or providers, both studies used blinded assessors. PERIOP-PC was a multisite trial that randomized patients with upper gastrointestinal cancers to either usual management with encouragement to follow National Comprehensive Cancer Network (NCCN) recommendations for PC referral or perioperative co-management by their surgical team and PC. Among 359 included patients, 11% in the surgeon-alone and 90% in the surgeon-PC co-management groups received PC. There was no significant difference in the primary outcome of 3-month health-related QOL as measured by the Functional Assessment of Chronic Illness Therapy–Palliative Care (FACIT-Pal) Subscale (control 138.54 vs. intervention 136.90; *p* = 0.62). While not statistically significant, both FACIT and PROMIS measures for the control and intervention groups decreased from baseline directly after surgery and recovered to baseline by the end of the study period.^33^

The SCOPE trial was a single-institution trial that similarly randomized patients undergoing major abdominal cancer surgery to either usual care or PC consultation preoperatively, during their hospital stay after surgery and postoperatively in the outpatient setting. Among 235 patients, 97% of patients assigned to the intervention arm were seen by PC specialists compared with 1% of patients in the usual care group. No differences were observed in the primary endpoint of physical and functional QOL at 90 days as measured by FACT-G TOI (control 45 vs. intervention 45; adjusted odds ratio [OR] 1.17, 95% confidence interval [CI] 0.77–1.80; *p* = 0.46). Secondary endpoints and exploratory outcomes, including anxiety (OR 0.98, 95% CI 0.65–1.50; *p* = 0.94), days alive at home (OR 0.89, 95% CI 0.69–1.11; *p* = 0.26) , overall survival (hazard ratio [HR] 0.97, 95% CI 0.50–1.88; *p* = 0.92), and caregiver burden (OR 0.97, 95% CI 0.61–1.53; *p* = 0.90), were also unchanged. ^34^

### Non-Randomized Studies

Non-randomized studies relevant to the role of PC in surgical oncology included two physician surveys, four retrospective cohort studies, and a prospective cohort study in which participants were assigned to treatment arms according to their preference. Physician surveys were conducted to gather insight from the provider’s perspective on PC referral. Adolfsson et al. surveyed Swedish physicians regarding their attitudes and practices surrounding the integration of oncology and PC. Findings showed that the vast majority (99.2%) of medical and surgical oncologists believed that introducing their patients to the concept of PC was beneficial, while 65% saw value in early PC consultation. Notably, medical specialties tended to place early PC referrals significantly more often than their surgical counterparts.^21^ Demyan et al. conducted an international survey of surgeons’ opinions on the utility of preoperative PC referral, of which 65% were in favor of introducing PC services in the preoperative setting, with female and fellowship-trained surgeons significantly more likely to support this practice.^22^

Several cohort studies were identified examining PC referral patterns for various patient populations undergoing oncologic surgery. One study was conducted to determine the factors that influence PC consultation in patients receiving cytoreductive surgery with hyperthermic intraperitoneal chemotherapy, a major abdominal operation. Overall, only 23% of patients were referred to PC, with older age and higher comorbidity associated with increased likelihood of PC consultation.^26^ In a separate study, timely PC referral was also noted to be infrequent among patients originally intended to undergo curative-intent surgery that had ultimately been unexpectedly aborted. In this cohort, only 34% of patients were seen by PC overall, and only 13% received PC consultation within 30 days of their aborted operation.^25^

A study investigating health disparities among breast cancer patients revealed that while traditional markers of poor healthcare access, such as minority race, lack of health insurance, low rates of high-school graduation, and poor median income, were associated with increased referral to ancillary services including PC, actual utilization of these services was lower for patients fitting these demographics.^24^ Furthermore, only two-thirds of referred patients attended an initial consultation.

Meanwhile, a non-randomized prospective cohort study performed in Taiwan demonstrated several benefits with early PC consultation in patients diagnosed with head and neck cancers, the majority of whom (78.3%) underwent curative surgery.^32^ In this study, patients voluntarily elected to receive early PC consultation or standard care. Results showed that early PC correlated with improved symptom control, reduced distress and depression, and enhanced QOL for patients with both early- and advanced-stage head and neck cancers. The authors felt that PC consultation allowed care teams to provide a more holistic approach to patient care, taking into consideration patients’ physical, nutritional, psychosocial, and financial needs.

Additionally, incorporation of a PC team into the workflow at a surgical oncology clinic was demonstrated to facilitate PC consultation and advanced care planning discussions prior to surgery. An increase in the advance directive designation rate from 72% to 85% was observed with PC integration, which was also associated with increased administration of palliative-intent therapies.^23^

### Qualitative Studies

One qualitative study was conducted to evaluate surgeons’ views towards PC consultation.^27^ The other four were nested within the two RCTs mentioned previously.^28–31^ Hartman et al. highlighted how surgeons and PC specialists differ in their appraisals of the role of PC among patients undergoing curative-intent cancer surgery. Most surgeons (77%) endorsed PC consultation, with 36% feeling strongly positive regarding its utilization and 41% feeling open to the idea with some reservations, compared with 100% of PC physicians. While 88% of surgeons reported feeling comfortable having end-of-life discussions, only 48% routinely had these conversations with patients in a preoperative setting. Lack of time or opportunity (86%), not wanting to take away hope before surgery (74%), and consideration of patient comfort (51%) were cited as reasons not to do so.^27^

A qualitative study nested within the PERIOP-PC trial identified five themes that characterized cancer patients’ experience with PC: (1) in general, patients typically had limited prior awareness or understanding of PC as a specialty; (2) patients perceived PC interventions as talking; (3) some patients whose concerns aligned with PC described it as having a positive impact on their experience; (4) most patients expressed a desire to focus on cure from their cancer instead; and (5) integrating specialist PC practitioners with surgical teams made it difficult for some patients to identify team member roles.^28^ The last three qualitative studies examined notes and interviews from surgical oncology patients enrolled in the SCOPE trial to determine their supportive care needs. Overall, patients undergoing cancer surgery commonly reported having at least one psychosocial stressor surrounding their operation and emphasized the importance of timely care and high-quality communication with their providers.^29–31^ While surgical patients seldom described having serious concerns regarding end-of-life issues, they did express appreciation for the supportive presence offered by PC specialists.^29–31^

### Quality Assessment

Of the 14 studies included in this review, 11 were high quality, 2 were medium quality, and 1 was low quality (electronic supplementary Table 1). The study rated as low quality and one of the studies of medium quality were published only as abstracts, and therefore their assessment was limited by the information available in the shorter format. Other qualitative studies were most commonly limited by not acknowledging the impact of culture, theory, or positionality of the researcher on the results. For both RCTs, lack of participant and investigator blinding was the most common reason for deductions in quality rating.

## Discussion

This systematic review summarizes the existing literature on the role of PC in surgical oncology, highlighting the unique considerations and unmet needs of surgical patients that differ from those with advanced cancer. Unlike advanced cancer populations, for whom early and routine PC referral has demonstrated clear benefits to QOL and symptom control, evidence for PC in the context of patients undergoing curative-intent surgeries remains inconclusive. Both the PERIOP-PC and SCOPE RCTs found no significant benefit with routine perioperative PC consultation on postoperative QOL among patients undergoing major abdominal cancer surgery.^33,34^ These findings underscore the need to clarify how and when PC is most optimally introduced within a surgical context, as well as to identify which particular subgroups of patients may derive the most benefit from their early involvement.

Based on evidence of improved outcomes with early integration of PC, the American Society of Clinical Oncology (ASCO), NCCN, and European Society for Medical Oncology (ESMO) all recommend early involvement of PC.^1,5,6,8–13^ More recent recommendations endorse earlier referral independent of cancer stage, and a 2024 update to ASCO guidelines has stated that PC should occur alongside active cancer treatment;^10,35^ however, whether these recommendations should apply to early-stage patients with curable malignancies remains unknown. Indeed, patients who are eligible for curative-intent surgery generally have early-stage or resectable cancers and may perceive PC to be confusing, irrelevant, or premature, viewing it as incompatible with their goal of cure. This tension was noted in multiple studies, including qualitative assessments nested within the PERIOP-PC trial.^28^ Nevertheless, surgical oncology patients often face challenges and stressors specific to their surgical experience, including postoperative pain, psychosocial concerns, and the physical demands of recovery.^30,36^ These findings indicate an opportunity to emphasize the role of PC in enhancing recovery and QOL, regardless of curative intent, to encourage timely uptake, and broader acceptance in the surgical setting by both patients and providers.

Although the PERIOP-PC and SCOPE trials reported negative results with PC intervention, several qualitative analyses highlighted ways in which surgical oncology patients may benefit from PC consultation.^29–31^ This suggests that further research is needed to identify subpopulations within surgical oncology for whom PC referral would be the most useful. Referral should be based on patient need and risk profile, rather than a uniform ‘one size fits all’ approach. For instance, surgical patients with high preoperative symptom burden or those undergoing high-risk procedures may represent ideal candidates for early PC intervention to prevent symptom escalation, manage expectations, and support postoperative recovery. Aborted curative-intent surgeries represent another area of particular vulnerability within surgical oncology. Patients have an unmet need for rapid and compassionate support to manage the heightened emotional and physical distress they may experience in these circumstances. The absence of timely PC consultation for these patients and difficulties with care coordination suggest an urgent need to establish PC protocols that address unplanned or unsuccessful operations.^25,37–39^ Finally, underutilization of PC has been documented among patients from disadvantaged backgrounds.^24,40^ Identifying the socioeconomic barriers that patients may face to receiving PC services and integrating PC referral with curative treatment plans could combat such issues with access.

Despite the positive opinion most surgeons have towards PC referral, both structural and cultural challenges exist to PC integration within surgical oncology.^21^ Structural barriers identified in physician surveys include limited time for preoperative discussions regarding PC, and constraints within care settings may not prioritize PC consultation for patients expected to have curative outcomes. Culturally, surgeons reported concerns that early PC referral may undermine patients’ optimism about surgical outcomes. Taken together, these findings suggest that primary PC delivered by patients’ surgical oncologists may represent an alternative and opportune model for meeting the supportive care needs of patients with early-stage cancers. Indeed, an alternative explanation for the negative results of the PERIOP-PC and SCOPE trials is that academic surgeons participating in the RCTs were effective at providing the essential elements of primary PC to those randomized to the control arms; however, most surgeons receive limited training in PC competencies, suggesting the need for further research and pragmatic trials.^35^ The development of surgeon-targeted PC training programs could help bridge the gap in surgeon-delivered palliative support and increase awareness of when specialist PC referral may be indicated.^41^

This review was limited by the small number of included studies, as well as the lack of shared endpoints. Nevertheless, several conclusions can be made regarding existing literature on the role of PC involvement in surgical oncology. Based on two large RCTs, the routine use of perioperative PC among all cancer patients undergoing major abdominal curative-intent cancer surgery is not supported. Future research on PC interventions should aim to target high-risk subpopulations, equip surgeons with the skills to solicit patients’ PC needs, and develop innovative methods of delivering multidisciplinary PC. Given existing literature on barriers to PC, additional research should also focus on educational efforts for both patients and providers on the purpose of PC and the role it may play in their treatment. These strategies, along with system-level changes to support PC integration, could pave the way for a more personalized and effective approach to PC in surgical oncology, ultimately fostering a culture of care that emphasizes QOL and patient well-being alongside curative goals.
